# The Evolving Role of the Oncology Nurse in the United States of America—A Survey Exploring Their Perspective

**DOI:** 10.3390/healthcare12232453

**Published:** 2024-12-05

**Authors:** Nina N. Grenon, Karen S. Waldrop, Natasha Pinheiro, Brittni Prosdocimo

**Affiliations:** 1 Dana Farber Cancer Institute, Boston, MA 02215, USA; 2 O’Neal Comprehensive Cancer Center, University of Alabama, Birmingham, AL 35233, USA; kswaldrop@uabmc.edu; 3 Memorial Sloan Kettering Cancer Center, New York, NY 10065, USA; pinheirn@mskcc.org; 4 UPMC Hillman Cancer Center, Pittsburgh, PA 15232, USA; bittnerb@upmc.edu

**Keywords:** oncology nurse, survey, role evolution, telehealth

## Abstract

Background: The role of the oncology nurse has evolved since the COVID-19 pandemic to continuously meet patient needs, resulting in an increased virtual presence. However, there is little information about whether these roles have continued since the pandemic and how it is affecting nurses. Methods: The aim of this study, conducted via an electronic survey questionnaire, was to examine the perspective of oncology nurses in the United States of America regarding how their role has changed, the adaptation to telehealth, challenges, and needs. Results: Over 5 months, 197 respondents from 17 different states completed the survey. They were all registered nurses working in oncology with different roles, such as being nurse practitioners (23%), nurse navigators (20%), infusion nurses (23%), and working in outpatient settings (93%). The findings of the survey highlight the multifaceted responsibilities of nurses in providing care across the cancer care continuum, emphasizing patient-centered care, communication, education, and empowerment, in addition to expected duties such as the administration of anticancer therapy, monitoring of side effects, and symptom management. A total of 82.6% of United States of America oncology nurses feel their workload has increased. The role of nurses in oncology is continuously evolving and the impact of the COVID-19 pandemic, in certain areas such as telehealth, is here to stay. Conclusions: The results of the study allow a better understanding of the emergent roles of oncology nursing. The importance of self-care initiatives and education are emphasized as critical to support nurses in their complex, busy, and emotionally charged work environment and to help retain existing nurses and attract new individuals into the profession. The study ultimately seeks to inform policy and drive professional development in oncology nursing in the United States of America.

## 1. Introduction

Nursing in the United States of America (USA) is a multilevel profession that includes registered nurses (RNs) and advanced practice registered nurses (APRNs), who have a Master’s or Doctorate degree in nursing [1,2]. Oncology nurses are highly qualified, expert, compassionate, and competent professionals. They support the multidisciplinary team to deal with difficult workloads, while at the same time being the most accessible to patients and their families among all the involved healthcare professionals [2,3].

Nurses working in the oncology setting hold many cross-functional responsibilities across the cancer care continuum, from health maintenance and disease prevention to survivorship and end-of-life [4,5]. This care continuum has the cancer patient and caregiver at its core (people-centered care) and requires effective communication between patients and all other involved healthcare professionals across hospital and community settings [5,6]. Nurses also provide patient- and family-focused education about disease, treatment, and symptom management. The roles in oncology nursing have evolved from being task-oriented to being increasingly specialized, including nurses as integral members of multidisciplinary teams, all driving quality in cancer care [3,7,8].

Considering the increasing burden of cancer in the USA and globally, and the ever-changing clinical management landscape, oncology nursing may be exceptional in its broad scope and set of responsibilities [9]. Most recently, the COVID-19 pandemic, the burden of which was unprecedented, resulted in rapid changes in the delivery of care and caused great demands on both patients and providers [10,11]. The prioritization of COVID-19-related care led to a disruption in cancer care services due to the redeployment of staff, pauses, and/or delays in screening/diagnostic tests and medical/surgical procedures. Importantly, COVID-19 infection and the physical and mental health exhaustion of cancer healthcare workers led to staff shortages [10,11,12]. Early post-pandemic studies highlight that COVID-19 has certainly reshaped oncology nursing [13,14]. One such area is that of telemedicine and telehealth. The use of these services by oncology nurses has been shown to be beneficial for patient outcomes [15]. Previously reported studies have shown how nurses were able to provide quality care during the pandemic by adapting their role as necessary but have not looked at how it has continued to shape the role of the oncology nurse and the support required for them to continue to expand their roles.

The aim of this study was to explore how the role of oncology nurses in the USA is continuously changing and to evaluate this from the nurses’ perspective. Although it was not intended to examine the effects of the COVID-19 pandemic per se, the survey aimed to understand how changes made during the pandemic may have irreversibly altered aspects of nursing. The survey focused on aspects related to the nature of nurses’ interactions with cancer patients, workload shifts, and the adoption of new technology.

## 2. Materials and Methods

### 2.1. Survey

The survey was designed in English and pre-tested with the assistance of the authors, who are oncology nurses (including two nurse practitioners, one oncology nurse navigator, and one advanced clinical nurse specialist), to ensure questions were clear. The survey questionnaire is provided in Appendix A. The initial part collected demographic information, followed by a section of questions exploring respondents’ opinions on the role of oncology nurses in treatment and symptoms management and the evolution of relevant responsibilities over the years; it also assessed oncology nursing practice changes since the COVID-19 pandemic. In addition, a qualitative section gathered feedback about nurses’ perceptions of the immediate future of their profession.

### 2.2. Ethical Considerations

The survey was confirmed to align with the Category 2 exemption of the Institutional Review Board (IRB) guidelines, as it was an anonymous, questionnaire-based research project that did not involve patient or confidential patient data. This category typically encompasses studies involving educational tests, surveys, interviews, or observations of public behavior that are either anonymous or pose minimal risk to respondents if the information were to be disclosed. The scope of the survey was confined to exploring the professional roles of oncology nurses in the US without collecting identifiable data, thus adhering to privacy and confidentiality standards.

Informed consent was obtained at the beginning of the survey for each participant, ensuring that respondents were aware of the study’s purpose, their voluntary participation, and the confidentiality of their responses.

### 2.3. Respondents and Procedures

Nurses practicing in various oncology care settings throughout the USA were invited to participate in the survey. They were categorized by specialization (further information on group definition is provided in Appendix B), practice type, the patient population they care for, experience, and geographic (state) location. The sample size was not pre-specified. The survey was conducted over a period of 6 months from January to June 2024 using the online platform Qualtrics XM (available from https://www.qualtrics.com/ (accessed on 10 July 2024)) and was distributed via email to nurses across professional organizations, networks, and various institutions through social media channels and the Scientific Committee. Due to the methods of distribution of the survey, it was not possible to determine response rates, i.e., how many nurses filled in the questionnaire out of the total number of nurses who received it.

### 2.4. Statistics

Descriptive statistics were used to report respondents’ responses with categorical variables presented as absolute (N) and relative (%) frequencies. Data elements that contained a proportion (e.g., % of respondents) were subjected to pairwise, two-tailed Z-Test analysis, yielding a *p*-value. We used a confidence level of 95%; therefore a *p* < 0.05 means there is more than a 95% chance that the difference seen was a real effect in the underlying population, not just due to the sample used in the study. The analysis was performed by an external company.

### 2.5. Open-Ended Questions

Open-ended questions were utilized to elicit perceptions of the evolving role of oncology nurses within the USA at present and in the next several years. The feedback was qualitatively examined and analyzed thematically in a systematic manner. The data were coded inductively and then organized to search for themes using Microsoft Word. Emerging themes were reviewed by the wider project team [16].

## 3. Results

### 3.1. Respondent Nurses’ Demographics

**Figure 1:**
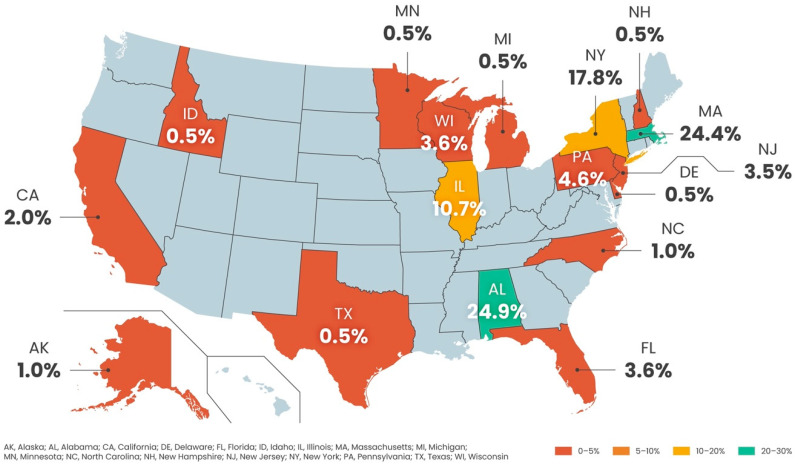
Geographical distribution of survey respondents across the United States.

A total of 197 respondents completed the survey between January and June 2024. The demographics of the respondents are presented in Table 1, and their geographical distribution is presented in Figure 1. There was representation from 17 different states across the country but most of the respondents came from three states (Alabama: 24.9.%, Massachusetts: 24.4%, and New York: 17.8%), possibly influenced by the members of the study’s Steering Committee.

The respondents were all registered nurses working in an oncology setting and representing different nursing roles, spanning the whole cancer care continuum. They included nurse practitioners (23%), nurse navigators (20%), infusion nurses (23%), oncology nurses (16%), registered nurses (other) (14%), and others (4%). The survey was completed by a highly qualified and experienced group of nurses, with more than one-third having over 10 years of nursing experience (34%) and the majority (a combined 53%) having a Master’s or Doctoral degree.

The majority of nurses (93%) indicated that they were working in outpatient settings, with most working in outpatient oncology clinics (63%), followed by those working at cancer centers designated by the National Cancer Institute (NCI) (34%). Some respondents selected two or more practice types.

### 3.2. Oncology Nurses’ Role in Managing Treatment and Its Effects

**Figure 2:**
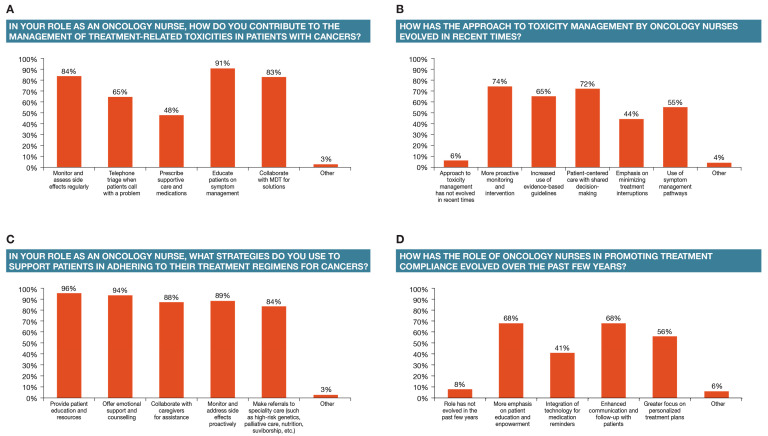
Role in managing treatment effects and its evolution.

The vast majority of the respondents affirmed the notion that their role is to educate patients on symptom management and monitor and assess side effects regularly; in this way, they collaborate with multi-disciplinary teams to plan tailored interventions to meet the patient’s unique needs (91%, 84%, and 83%, respectively) (Figure 2A).

A significantly greater proportion of infusion nurses, RNs (other), and nurse practitioners monitor and assess side effects regularly compared to nurse navigators (*p* < 0.05). Also, a significantly greater proportion of nurse practitioners perform proactive monitoring and intervention than nurse navigators or oncology nurses (*p* < 0.05). In contrast, and as may be expected, a significantly larger proportion of all other groups are more involved in telephone triage when patients call with a problem than infusion nurses (*p* < 0.05).

When asked about how the management of treatment toxicities has evolved, the survey respondents commented that it has become more proactive in terms of monitoring and intervention (74%), there is more patient-centered care with shared decision-making (72%), increased use of evidence-based guidelines (65%), use of symptom management pathways (55%), and more emphasis on minimizing treatment interruptions (44%) (Figure 2B). Only a small fraction of the respondents (6%) thought that the approach to toxicity management has not evolved in recent times. A significantly larger proportion of nurses with more than 10 years of experience reported an increased use of evidence-based guidelines when compared to those with fewer years of experience (*p* < 0.05).

A critical aspect of ensuring benefits from anticancer therapy and the management of toxicities is the monitoring and promotion of adherence to treatment plans [17]. Survey respondents answered that they use different strategies to promote adherence, including providing patient education and resources (96%), offering emotional support and counseling (94%), monitoring and addressing side effects proactively (89%), collaborating with caregivers for assistance (88%), and making referrals to specialty care (84%) (Figure 2C).

A high proportion of respondents considered that promoting treatment compliance has evolved in recent times–68% indicated that there is more emphasis on patient empowerment and communication and follow-up with patients, 65% noted a greater focus on personalized plans, and 41% noted the integration of new technology. Similarly, an evolution in terms of the overall management of anticancer treatment and its toxicities is perceived to be happening–74% reported more proactive monitoring and intervention, 72% reported more patient-centered care with shared decision-making, and 65% reported increased use of evidence-based guidelines. Finally, survey respondents indicated that the role currently places more emphasis on patient empowerment (68%), enhanced communication, and follow-up with patients (68%) than in the past. (Figure 2D).

### 3.3. Oncology Nurse’s Role in Assessing and Managing Quality-of-Life

**Figure 3:**
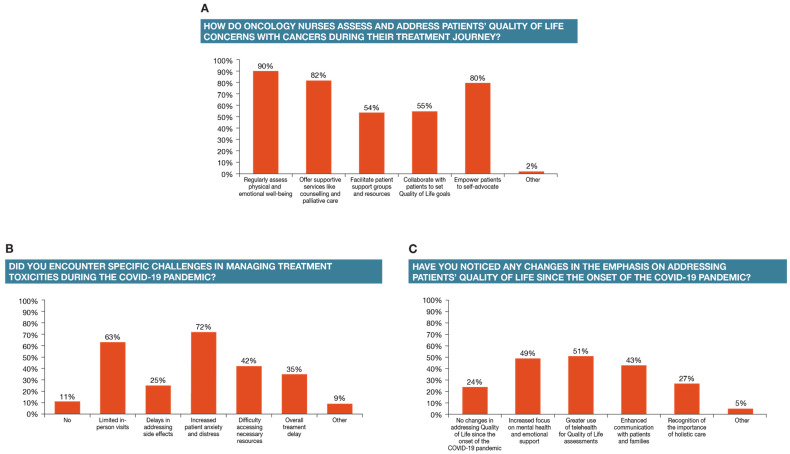
Role in managing treatment effects and QoL—evolution post COVID-19 pandemic.

The assessment and management of quality-of-life (QoL) concerns during treatment is considered an integral part of oncology nurses’ role in managing therapy administration and its side effects [5,6,7]. The survey respondents responded that they regularly assess physical and mental well-being (90%), offer supportive services like counseling and palliative care (82%), collaborate with patients to support QoL goals (55%), empower patients to self-advocate (80%), and facilitate patient support groups and other services (54%) (Figure 3A). A small proportion (2%) indicated other activities, including discussing advanced care planning and what is important to the patient and family. A significantly greater proportion of nurse practitioners, nurse navigators, and RNs (other) offer supportive services like counseling and palliative care compared to infusion nurses (*p* < 0.05 in all comparison categories versus infusion nurses). As may be expected, a significantly greater proportion of nurses with more than one year of experience offer emotional support and counseling compared to those with less than one year of experience (*p* < 0.05 for each experience category versus less than one year of experience).

The survey respondents were also asked about the challenges they faced in managing treatment toxicities during the COVID-19 pandemic and whether addressing QoL issues may have changed since the pandemic. The respondents affirmed that increased patient anxiety and distress (72% of the respondents) and limited in-person visits (63% of the respondents) were seen during the pandemic. A lower proportion reported having difficulty in accessing necessary resources (42%), having treatment delays (35%), and delays in addressing side effects (25%), whereas 11% reported facing no challenges (Figure 3B). Despite the challenges, positive changes in addressing QoL since the pandemic were noted, such as a greater emphasis on the use of telehealth for QoL assessments (51%), increased focus on mental health and emotional support (49%), and enhanced communication with patients and families (43%). However, approximately a quarter of the respondents (24%) reported having seen no changes in how QoL issues are addressed since the onset of the pandemic (Figure 3C). 

### 3.4. Oncology Nurses’ Role as an Educator

**Figure A1:**
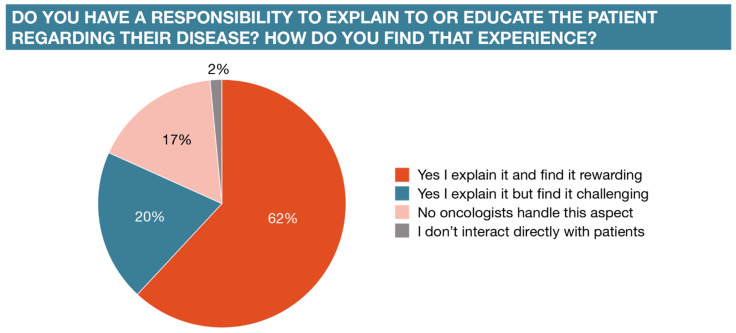
Oncology nurse’s role as an “educator”.

Beyond managing treatment and side effects, nurses in oncology settings are required to have a deep understanding of the natural history and clinical features of the patient’s cancer [3,5]. Oncology nurses must be knowledgeable about appropriate procedures and treatments, their expected toxicity, and the symptoms of advancing disease, and they should educate patients and family members accordingly. When asked about their responsibility for explaining/educating patients and how they find the experience, a large proportion of survey respondents (62%) responded that they educate patients regarding their disease and find such activities rewarding but 20% commented that they find it challenging. On the other hand, 17% responded that oncologists alone handle such aspects of patient education (Figure A1).

The majority of nurse navigators (87%) responded that they have a responsibility to educate patients regarding their disease and find this rewarding, followed by nurse practitioners (74%) and (other) RNs (67%). The highest proportion of those reporting that they have a responsibility to educate patients regarding their disease but find it challenging were oncology nurses (32%), followed by infusion nurses (29%).

The proportion of nurses answering “Yes, I explain it and find it rewarding” increased with the years of experience from 33% in those with less than one year of experience to 48%, 71%, and 77% in those with 1–5, 5–10, and over 10 years, respectively. In contrast, for those answering “Yes, I explain it but find it challenging”, the proportion decreased with experience.

### 3.5. Oncology Nurses’ Participation in Treatment Decision-Making

**Figure A2:**
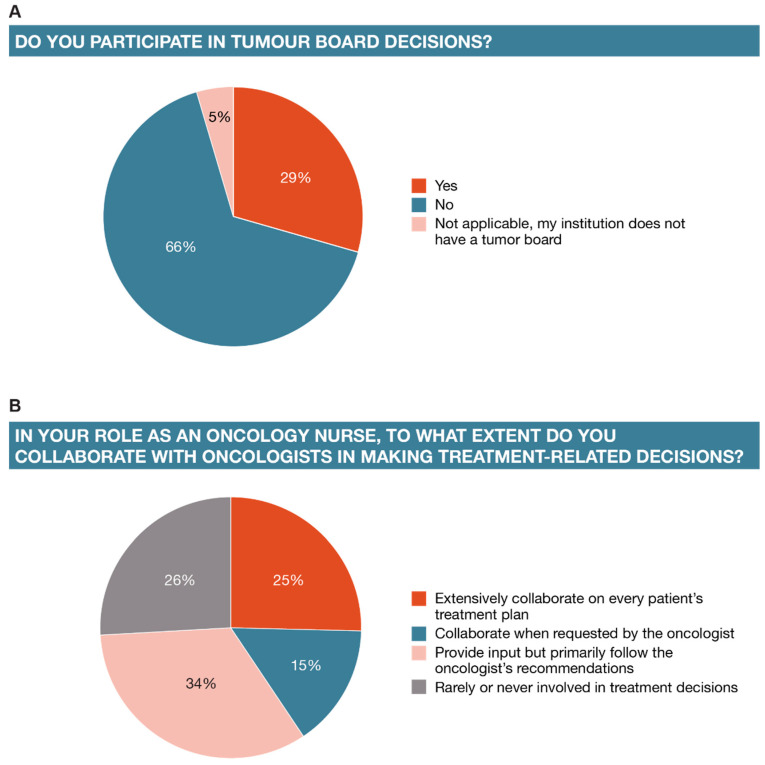
Oncology nurses’ participation in treatment decision-making.

Being an active part of multidisciplinary teams is essential to the role of oncology nurses in effectively supporting their patients [3,18,19]. This also implies being a member of the Multidisciplinary Tumor Board (MTB). Strikingly, 66% of the survey respondents responded that they do not participate in MTBs, with only 29% confirming they do participate (Figure A2). Among the respondents, the highest proportion of nurses participating in MTBs were nurse practitioners (63%) and nurse navigators (44%). Infusion nurses (100%) and a large majority of the (other) RNs (89%) responded that they do not participate in MTBs. The proportion of nurse practitioners reporting participating in MTBs was statistically significantly higher (*p* < 0.01) compared to that of infusion nurses, oncology nurses, or (other) RNs. The same was true for nurse navigators compared to infusion nurses and or (other) RNs (*p* < 0.01). The level of experience also appears to correlate with involvement, with 40% of those with 5–10 years’ experience reporting that they are involved in MTBs, which is more than those with less than 5 years of experience (29% if 1–5 years of experience, 11% if less than 1 year of experience), although that changes with only 27% of those with over 10 years of experience being part of the MTBs.

Similarly, the collaboration and involvement of oncology nurses in treatment decision-making appears to be low, with 34% of respondents indicating that they provide some input but primarily follow the oncologist’s recommendations and 26% saying that they are rarely or never involved in treatment decisions. A quarter (25%) of the respondents, however, reported that they collaborate extensively on every patient’s treatment plan. Among nurses with over 5 years of experience, approximately two-thirds reported that they extensively collaborate with oncologists in making treatment-related decisions: 31% and 30% of nurses with 5–10 and more than 10 years’ experience, respectively. Only 17% and 22% of those with 1–5 years and less than 5 years, respectively, reported the same. The survey results showed that a significantly greater proportion of nurse practitioners collaborate on every patient’s treatment plan more than the other nurse groups (*p* < 0.05). Infusion nurses had the highest proportion of respondents who were rarely/never involved with treatment decision-making, with only 2% indicating that they extensively collaborate or collaborate when requested by the oncologist.

**Figure 4:**
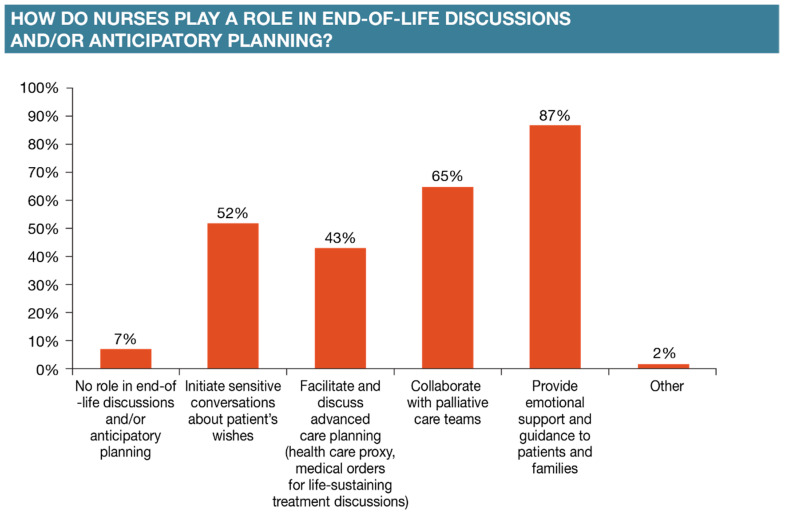
Oncology nurses’ participation in end-of-life decisions and planning.

Notwithstanding, a significantly greater proportion of oncology nurses responded that they are much more engaged in end-of-life discussions and/or participatory planning (*p* < 0.05 versus nurse navigators, infusion nurses, and oncology nurses). The majority of the survey respondents (87%) indicated that they provide emotional support and guidance to patients and families, collaborate with palliative care teams (65%), initiate sensitive conversations about patients’ wishes (52%), and facilitate and discuss advanced care planning (43%) (Figure 4).

### 3.6. Oncology Nurses’ Role Post-Pandemic

**Figure A3:**
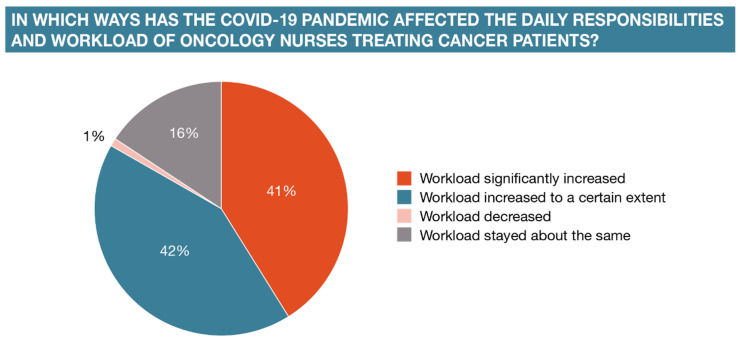
Oncology nurses’ workload during the COVID-19 pandemic.

A key aim of the study was to understand the long-term, irreversible impact (negative or positive) of the COVID-19 pandemic on the role of oncology nurses and possible changes to their clinical practices. When asked about the effects of the pandemic on daily responsibilities and workload, 83% of the respondents reported that the workload increased either to a certain extent (42%) or significantly increased (41%) (Figure A3). This was mostly the case for RNs (other), nurse practitioners, and infusion nurses, with 96%, 89%, and 82%, respectively, responding that their workload significantly increased.

**Figure A4:**
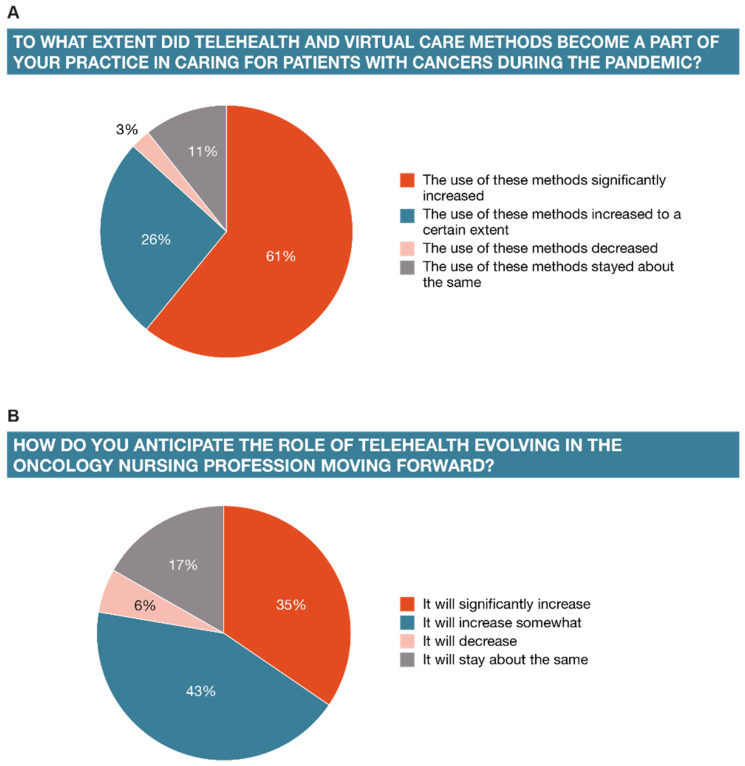
The use of telehealth and virtual healthcare tools during the COVID-19 pandemic and moving forward.

The survey inquired specifically about the utilization of telehealth and virtual healthcare tools during the pandemic and their potential impact on oncology nursing practices. According to the respondents, the use of such methods significantly increased (61%) or increased to a certain extent (26%), with a lower percentage of respondents perceiving that it stayed about the same (11%) or even decreased (3%). At the same time, most respondents believed that the use of telehealth and virtual care in oncology nursing will increase (78%). Among the respondents, 17% believed that it will stay about the same and 6% believed that it will decrease (Figure A4).

**Figure 5:**
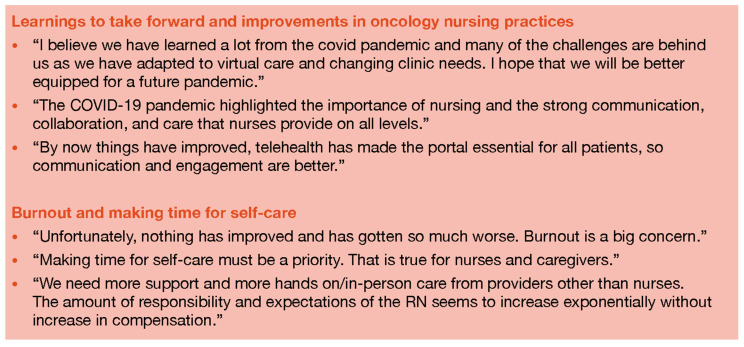
Oncology nurses’ remarks on shared pandemic experiences.

Qualitative feedback provided in the survey concerning the experiences of nurses caring for cancer patients during the pandemic is summarized in Figure 5. The study results are ambiguous as to whether the working environment of nurses in oncology has become better or worse pre-, during, and post-COVID-19 pandemic. Some respondents perceived that it has worsened but others considered that things are becoming better compared with how challenging they were during the pandemic. Importantly, however, respondents indicate that there is a lot to be learned from such an experience. Particularly, strong collaboration and communication between different healthcare providers and disciplines, and certain practices, including the use of telehealth and other virtual healthcare tools, are critically changing oncology and nursing in general in a positive direction.

There were strong remarks regarding burnout, the need for support, learning to cope with emotional distress, and making time for self-care. The respondents found it of paramount importance to recognize the additional effort and reward the extra responsibilities that have been imposed on oncology nurses to ensure job satisfaction and prevent nurses from abandoning the profession.

### 3.7. The Future Role of Oncology Nurses

The increasing use of telehealth, virtual clinics, and online apps to reduce the number of in-person patient visits were recurring themes of the qualitative data analysis. Nurse practitioners and nurse navigators perceive that this change will be especially popular in ambulatory clinics. In contrast, infusion nurses expressed the opinion that virtual care is not an option for them, given the nature of the care they deliver.

**Figure 6:**
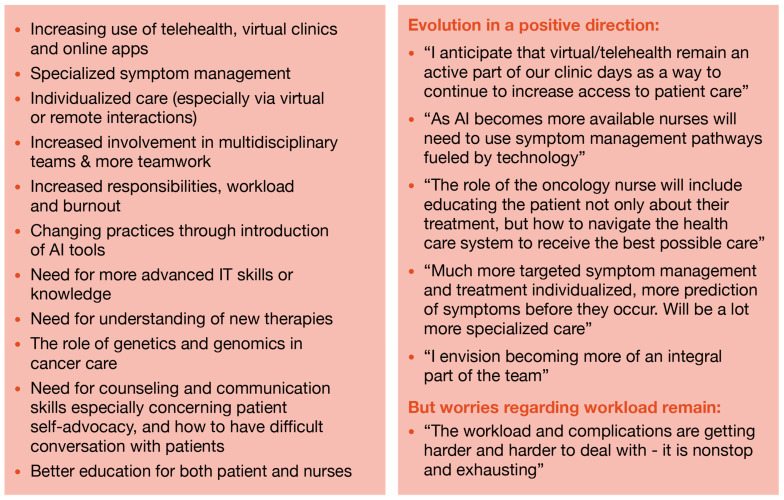
Emerging themes regarding the future of the role of oncology nurses.

A considerable proportion of the respondents reported they expect oncology nurses to become more involved in patients’ education and providing patient and family support. Some also mentioned the increasing importance of oncology nurses as educators for patient self-advocacy and the need for improved counseling and communication skills, especially when dealing with difficult conversations with patients. The need for continuing and improved education for both oncology nurses and patients were highlighted, especially regarding knowledge about new therapies (e.g., novel targeted and Chimeric Antigen Receptor (CAR)-T cell therapies) and more advanced information technology (IT) skills. Some respondents also found it important to improve knowledge in areas such as the application of genetics, genomics, and artificial intelligence (AI) tools in cancer care (Figure 6).

Finally, respondents frequently mentioned worries that they predicted the increased responsibilities of oncology nurses would increase rates of burnout and in turn lead to fewer people wanting to enter the profession.

## 4. Discussion and Conclusions

The results of this study affirmed the multifaceted nature of the role of oncology nurses in the USA. The participating nurses perceive that their central role is managing treatment-related toxicity and disease-related symptoms by consistently monitoring patients. They believe that education is a key aspect of patient care and their role also includes advocating for their patients and providing emotional support. It is also clear that the survey respondents perceive that their role has changed and evolved in recent times. Oncology nurses are being given more responsibilities, are increasingly more proactive and more involved in multidisciplinary teams, and are using more technology in their day-to-day work. An evolution has also occurred from a conceptual perspective, meaning that there is an increasing emphasis on a patient-centered approach and more patient advocacy by empowering cancer patients to be more involved in the decision-making about their care and treatment.

Oncology nurses are required to deal with unique physical symptoms related to the disease, the toxicity of the patients’ anticancer therapies, psychological distress, and QoL concerns. Moreover, there is the uncertainty of a cancer diagnosis and financial “toxicity” concerns that affect patients and their families [20]. Although providing high-quality, person-centered care is rewarding and affords oncology nurses immense satisfaction, several studies have found that occupational stress and burnout are prevalent workplace issues for oncology nurses [21,22]. This can be due to both organizational challenges and personal situations [23]. The findings of this study indicate a busy, if not heavy, workload. The emotional experience, heavy workload, and resulting distress underlie burnout, poor communication, the inability to provide compassionate care, and compassion fatigue [24,25,26]. Critically, oncology nurses are frequently exposed to loss and suffering, which they may be unprepared (untrained) or unable to cope with effectively [24]. Hence, implementing self-care strategies for understanding burnout and building emotional resilience is necessary [27].

Approximately two-thirds of all nurses surveyed in this study do not participate in Multidisciplinary Tumor Boards (MTBs), which, while disappointing, is not unexpected. Previous studies have also found that nurses are not involved in decision-making in oncology and other clinical settings [28,29,30]. This is despite the well-documented positive impact of clinical nurse specialists on the decision-making process in MTB meetings and improving overall patient care outcomes [31,32]. Different barriers may exist that do not permit the involvement of oncology nurses in treatment decision-making [32]. Previous studies have shown that the workload, time constraints, a lack of confidence in their skills, and misconceptions on the part of physicians or nurses themselves may explain the lack of participation [30]. These factors need to be understood and overcome, since beyond planning and managing cancer therapy, it is most often oncology nurses who best understand the social and personal situation of the patient and are the direct line of communication to coordinate their care [3,5].

A particular facet the survey sought to examine was the permanent impact of any changes introduced in oncology nursing practices during the COVID-19 pandemic. The survey confirmed how some changes incorporated during the pandemic have now become part of routine practice, especially concerning the use of telehealth and virtual care tools, but also highlighted the need for continuing education. The introduction of telehealth started long before the pandemic, and the “Telehealth in Oncology: American Society of Clinical Oncology (ASCO) Standards and Practice Recommendations,” by ASCO was made available in 2021 [33]. The findings from the present survey corroborate previous reports, both global and European, which found that the pandemic has ushered in changes for attending to and caring for patients with cancer, such as telehealth and the use of virtual tools, which are here to stay [34,35]. Interestingly, a recent study that evaluated patient and staff experiences of virtual cancer care at a large urban, tertiary cancer center in Canada reported that more patients than physicians were satisfied with virtual care. In addition, fewer patients than physicians perceived that virtual visits were worse than those conducted in person, and that telephone and video visits negatively affected human interaction [36]. In this same study, healthcare professionals demanded guidance.

Beyond telehealth, there were also references to the role of AI in oncology nursing in the respondents’ feedback to the open-ended questions. AI technologies have made rapid advances in cancer care; for example, genomic medicine and diagnostic support are already extensively used to perform gene and image analysis that supports disease management. The increasing role of AI tools, especially in early detection and screening in cancer care, was clearly noted. However, the involvement of cancer nurses in the development of AI in cancer care is limited [37,38]. The survey respondents commented that nurses need to learn and become comfortable with AI tools to improve patient outcomes,, increase satisfaction with care, and decrease costs. Many experts expect that AI will further continue to reshape the role of oncology nurses and be integrated into their work [39].

While nurses are leading changes and overcoming challenges, they are also requesting support to overcome some of the barriers. The need to enhance training and education in the immediate future was highlighted in the thematic analysis of the survey. As pointed out by other studies, this is specifically in areas such as precision medicine, genomics, and biomarkers. Oncology nurses will need to become more knowledgeable and continue learning about new cancer treatments with novel biological mechanisms of action and side effects [40,41,42]. Oncology is a specialized field of nursing practice that requires good knowledge and competence but different nursing roles in the USA have different access to education [8]. To reduce inequalities, institutions and oncology societies need to work collaboratively, like during the pandemic, by providing education and recommendations that are available to everyone, making sure healthcare providers have access to updated and secure systems, unifying systems so it does not depend on where you work, and designating leaders to create changes [34,35].

The need to improve communication and counseling skills accordingly was also remarked. Communication is a cornerstone of patient care. Oncology nurses need to become comfortable with discussing healthcare proxies and advanced care planning in general. It is within the scope of their practice to understand what is important and meaningful to patients. Consequently, it requires nurses to learn effective communication skills. This is particularly important in planning for end-of-life discussions and other sensitive topics [43]. Nurses will be increasingly expected to deliver primary palliative care in the future due to insufficient available palliative care specialists and an increase in the aging population [44]. This is particularly important in a role known to experience high levels of burnout and compassion fatigue [45]. Improving communication skills can directly impact patient care, improve interdisciplinary communication, and reduce compassion fatigue [46].

Learning from the experience of COVID-19, collaborative approaches for common challenges to gain more knowledge and adjust to new needs improve healthcare systems and support the workforce [5,47]

### Study Strengths and Limitations

The survey was conducted across a large number of states in the USA, covering different geographic regions, backgrounds, and a range of different types of healthcare institutions. It gathered feedback from nurses caring for patients with a range of cancer types and working in different units and departments, allowing for a broad range of experiences to be represented in the sample. Data analysis was broadly descriptive in line with the key aim of understanding beliefs, perceptions, and trends across the sample population, but did not make comparisons between institutions, geographies, or roles, which may be explored further in subsequent studies.

A key study limitation was the fact that while many respondents were from diverse backgrounds and institutions, not all states were represented. Future research would benefit from broader geographic inclusion.

In addition, only a limited number of community nurses and those from inpatient settings responded to the survey; while the survey reflects their perspective, additional insights from inpatient and community hospital settings would be valuable for a comprehensive understanding of oncology nursing roles.

The response rate was not captured. No information was collected about the reasons for non-participation in the survey.

It should also be noted that nursing job descriptions or titles are varied, and they may indicate similar but also different responsibilities. They may also reflect changes in the profession with, for example, nurse navigators being increasingly more common. Therefore, during the data analysis, nursing roles were grouped in such a way as to represent real-life clinical practice and allow meaningful and clinically relevant data interpretation.

Lastly, the survey was developed specifically for this study and included content and face validity. In the future, it is recommended that internal consistency and structural validity be established. Future research could consider standardized burnout measures or find a connection between the results and organizational dynamics or patient outcomes.

## 5. Conclusions

Highly educated oncology nurses are a core component in the delivery of effective supportive cancer care. Oncology nurses are and will be increasingly called upon to ensure timely access to appropriate care throughout the cancer care continuum. Their roles in the USA are expanding and evolving, making sure that new patients are seen in a timely fashion, have no barriers to accessing treatment, and have the support needed. Some changes have brought opportunities to improve patient care and make it more accessible, such as telemedicine. The introduction of new treatment concepts and technology is inevitable, but an effort is needed to decrease the workload and emotional demands that are currently affecting nurses. Therefore, enhanced education and training to prepare oncology nurses to meet these demands are urgently needed. Joint efforts from societies and institutions to work collaboratively and make existing education more accessible could improve this workforce’s current challenges.

Moving forward, oncology care will continue to be transformed by utilizing nurses for a patient-centered approach, helping patients navigate through the challenges of their cancer diagnosis journey. During the COVID-19 pandemic, we learned of the devastating impact that a delayed diagnosis can have. However, positive changes were introduced that are here to stay to ensure optimum care and positive outcomes for patients and their families.

## Figures and Tables

**Table 1:** Demographics of the survey respondents (N = 197).

Survey Respondent Demographics		%	NP (N = 46)	NN (N = 39)	IN (N = 45)	ON (N = 31)	GN (N = 27)
Gender	Female	96%	98%	95%	98%	97%	89%
	Male	4%	2%	5%	2%	3%	11%
Age	<30	16%	12%	5%	20%	33%	15%
	31–40	30%	35%	39%	22%	13%	44%
	41–50	21%	23%	24%	22%	17%	11%
	51–60	19%	19%	13%	20%	27%	15%
	>60	14%	12%	18%	16%	10%	15%
Educational background	College degree/nursing program	40%	15%	44%	53%	61%	52%
	Undergraduate degree	27%	13%	38%	38%	23%	33%
	Master’s degree	26%	46%	18%	9%	13%	15%
	Doctoral degree	7%	26%	0%	0%	3%	0%
Nursing role	Nurse practitioner	23%					
	Nurse navigator	20%					
	Infusion nurse	23%					
	Oncology nurse	16%					
	Registered nurse (other)	14%					
	Other	4%					
Number of years in current role	<1 year	9%	4%	10%	7%	6%	19%
	1–5 years	33%	5%	7%	9%	7%	5%
	5–10 years	24%	10%	5%	5%	1%	3%
	>10 years	34%	81%	78%	79%	86%	74%
Practice type	Academic/urban hospital	29%	30%,	31%	20%	39%	33%
	Community hospital	2%	4%	0%	2%	3%	0%
	National Cancer Institute	34%	39%	28%	20%	42%	33%
	Infusion center	20%	9%	10%	64%	0%	4%
	Academic institution	22%	28%	31%	9%	16%	22%
	Outpatient oncology clinic	63%	57%	72%	62%	52%	85%
	Other	1%	2%	0%	0%	0%	0%
Patient population setting	Inpatient	6%	2%	0%	0%	29%	0%
	Outpatient	93%	96%	100%	100%	71%	100%
	Both	1%	2%	0%	0%	0%	0%
Number of patients per week	1–5 patients	4%	0%	5%	0%	13%	4%
	6–10 patients	6%	2%	8%	4%	13%	0%
	11–15 patients	6%	11%	10%	0%	6%	0%
	16–20 patients	21%	20%	33%	20%	13%	22%
	>20 patients	63%	67%	44%	76%	55%	74%
Types of cancer	Colorectal	63%	54%	56%	87%	52%	63%
	Gastroesophageal	59%	57%	54%	84%	48%	44%
	Liver	50%	35%	49%	84%	32%	37%
	Pancreatic	61%	52%	56%	87%	45%	56%
	Neuroendocrine	54%	54%	46%	78%	35%	48%
	Other	12%	15%	13%	11%	6%	11%
	None	26%	37%	31%	9%	32%	26%

Abbreviation: IN, infusion nurse; NN, nurse navigator; NP, nurse practitioner; ON, oncology nurse; GN, registered nurse. Practice type and type of cancer %: respondent could select more than 1 option.

## Data Availability

The original contributions presented in the study are included in the article; further inquiries can be directed to the corresponding author/s.

## Appendix A

**Survey questionnaire** **Demographic Information** **Please indicate in which US state you currently practice** **Please state your age** - <30 - 31–40 - 41–50 - 51–60 - 61+ **Please state your gender** - Male - Female - Non-binary - Other **Please select your educational background in nursing:** - Technical degree/licensed practical program - College degree/diploma nursing program 1. Bachelor (BSN) 2. Associate degree (AD) 3. Diploma - Undergraduate degree (e.g., BA, BS in another field) - Master’s degree in nursing - Doctoral degree in nursing 4. PhD 5. DNP - Other (please specify) [Free text] **Please specify your role:** - Clinical nurse specialist - Clinical trial nurse - Radiation oncology nurse - Infusion nurse - Surgical oncology nurse - Nurse Practitioner - Oncology Nurse Navigator - Other (please specify) [Free text] **Please specify the number of years in your current role:** - <1 year - 1–5 years - 5–10 years - >10 years **Please specify your practice type (please select all that apply):** - Academic/Urban Hospital - Community Hospital - NCI (National Cancer Institute) designated Cancer Center - Infusion Center - Academic Institution - Outpatient Oncology Clinic - Other (please specify) [Free text] **Please specify your principal patient population:** - Inpatient - Outpatient - Other (please specify) [Free text] **Please specify the approximate number of patients who receive cancer treatment under your care per week:** - 1–5 patients - 5–10 patients - 10–15 patients - 15–20 patients - More than 20 patients **What types of gastrointestinal cancer do you treat? Select all relevant choices.** - Colorectal - Gastroesophageal - Liver - Pancreatic - Neuroendocrine - Other (please specify) [Free text] - I do not treat gastrointestinal cancers [EXCLUSIVE RESPONSE] **[Will not appear if participants select final option to Q8]** **Approximately, what percentage of the patients you see each year have gastrointestinal cancer?** - 0–10% - 10–25% - 25–50% - 50–75% - More than 75% **Section 1: Role in Treatment Management** **Treatment Selection Process & Treatment Adjustments:** **Do you participate in tumor board meetings?** - Yes - No - Not applicable, my institution does not have a tumor board. **In your role as an oncology nurse, to what extent do you collaborate with oncologists in making treatment-related decisions?** - Extensively collaborate on every patient’s treatment plan - Collaborate when requested by the oncologist - Provide input but primarily follow the oncologist’s recommendations - Rarely or never involved in treatment decisions - Prescribe supportive medication **Do you have a responsibility to explain to or educate the patient regarding their disease? How do you find that experience?** - Yes, I explain it and find it rewarding - Yes, I explain it but find it challenging - No, oncologists handle this aspect - I don’t interact directly with patients **Managing Treatment Compliance:** **In your role as an oncology nurse, what strategies do you use to support patients in adhering to their treatment regimens for cancers? Select all that apply** - Provide patient education and resources - Offer emotional support and counselling - Collaborate with caregivers for assistance - Monitor and address side effects proactively - Make referrals to specialty care (such as High-risk genetics, palliative care, nutrition, survivorship, etc.) - Other (please specify) [Free text] **How has the role of oncology nurses in promoting treatment compliance evolved over the past few years? Please consider both oral treatment and scheduling compliance. Select all that apply.** - Role has not evolved in the past few years [EXCLUSIVE RESPONSE] - More emphasis on patient education and empowerment - Integration of technology for medication reminders - Enhanced communication and follow-up with patients - Greater focus on personalized treatment plans - Other (please specify) [Free text] **Toxicity Management:** **In your role as an oncology nurse, how do you contribute to the management of treatment-related toxicities in patients with cancers? Select all that apply.** - Monitor and assess side effects regularly - Telephone triage when patients call with a problem - Prescribe supportive care and medications - Educate patients on symptom management - Collaborate with multidisciplinary teams for solutions - Other (please specify) [Free text] **How has the approach to toxicity management by oncology nurses evolved in recent times? Select all that apply.** - Approach to toxicity management has not evolved in recent times [EXCLUSIVE RESPONSE] - More proactive monitoring and intervention - Increased use of evidence-based guidelines - Patient-centered care with shared decision-making - Emphasis on minimizing treatment interruptions - Use of symptom management pathways - Other (please specify) [Free text] **Did you encounter specific challenges in managing treatment toxicities during the COVID-19 pandemic? If yes, select all that apply.** - No [PROGRAMMING–EXCLUSIVE OPTION] - Limited in-person visits - Delays in addressing side effects - Increased patient anxiety and distress - Difficulty accessing necessary resources - Overall treatment delay - Other (please specify) [Free text] **Quality of Life Management:** **How do oncology nurses assess and address patients’ Quality of Life concerns with cancers during their treatment journey? Select all that apply.** - Regularly assess physical and emotional well-being - Offer supportive services like counselling and palliative care - Facilitate patient support groups and resources - Collaborate with patients to set Quality of Life goals - Empower patients to self-advocate - Other (please specify) [Free text] **Have you noticed any changes in the emphasis on addressing patients’ Quality of Life since the onset of the COVID-19 pandemic? Select all that apply.** - No changes in addressing Quality of Life since onset of the COVID-19 pandemic [EXCLUSIVE RESPONSE] - Increased focus on mental health and emotional support - Greater use of telehealth for Quality-of-Life assessments - Enhanced communication with patients and families - Recognition of the importance of holistic care - Other (please specify) [Free text] **How do nurses play a role in end-of-life discussions and/or anticipatory planning? Select all that apply** - No role in end-of-life discussions and/or anticipatory planning [EXCLUSIVE RESPONSE] - Initiate sensitive conversations about patients’ wishes - Facilitate and discuss advanced care planning (health care proxy, medical orders for life-sustaining treatment discussions) - Collaborate with palliative care teams - Provide emotional support and guidance to patients and families - Other (please specify) [Free text] **Section 2: Evolution Since COVID-19** **Impact of COVID-19:** **In which ways has the COVID-19 pandemic affected the daily responsibilities and workload of oncology nurses treating cancer patients?** - Workload significantly increased - Workload increased to a certain extent - Workload decreased - Workload stayed about the same **Telehealth and Virtual Care:** **To what extent did telehealth and virtual care methods become a part of your practice in caring for patients with cancers during the pandemic?** - The use of these methods significantly increased - The use of these methods increased to a certain extent - The use of these methods decreased - The use of these methods stayed about the same **How do you anticipate the role of telehealth evolving in the oncology nursing profession moving forward?** - It will significantly increase - It will increase somewhat - It will decrease - It will stay about the same **Future Evolution:** **From your perspective, how do you envision the role of oncology nurses in the management of patients with cancers evolving in the next 2–3 years?** **[Free text]** **Are there any specific skills or knowledge areas that you believe will become increasingly important for oncology nurses in the near future?** **[Free text]** **Is there anything else you would like to share about your experiences as an oncology nurse in the context of managing patients with cancers and navigating the challenges brought about by the COVID-19 pandemic?** **[Free text]**

## Appendix B

**Definition of nurse groups for data analysis**

The survey was open to registered nurses working in an oncology setting, irrespective of specialized training, qualifications, or formal job title. It provided the following options for participants to select regarding their role: (1) Clinical nurse specialist, (2) Clinical trial nurse, (3) Radiation oncology nurse, (3) Infusion nurse, (4) Surgical oncology nurse, (5) Nurse Practitioner, (6) Oncology Nurse, (7) Navigator, and (8) Other (including a free text box for further specification).

During data analysis, the Steering Committee decided to combine and amend these groupings to reflect actual nursing practice in oncology based on the results received including the free text feedback. The revised groupings included 1) Nurse Practitioners (including both Clinical Nurse Specialists and Nurse Practitioners), 2) Nurse Navigators (including both Clinical Trial Nurses and Oncology Nurse Navigators), 3) Infusion Nurses (as above), 4) Oncology Nurses (including Radiation Oncology Nurses and Surgical Oncology Nurses along with those who had answered “Other” and specified in the text that they were Medical Oncology Nurses), 5) Registered Nurses (including those who had responded “Other” and specified in the text that they were Office Practice Nurses, Registered Nurses, Procedure Nurses or Clinic Nurses) and 6) Other (including Educators and Managers).
